# A knowledge base for *Vitis vinifera *functional analysis

**DOI:** 10.1186/1752-0509-9-S3-S5

**Published:** 2015-06-01

**Authors:** Alfredo Pulvirenti, Rosalba Giugno, Rosario Distefano, Giuseppe Pigola, Misael Mongiovi, Girolamo Giudice, Vera Vendramin, Alessandro Lombardo, Federica Cattonaro, Alfredo Ferro

**Affiliations:** 1 Department of Clinical and Experimental Medicine, University of Catania, Viale A. Doria 6, Catania, Italy; 2 Department of Computer Science, University of Verona, Strada le Grazie 15, Verona, Italy; 3 IGA Technology Services, J.Linussio, 51, Udine, Italy; 4 Department of Computer Science, University of Catania, Viale Andrea Doria, 6, Catania, Italy; 5 Cardiovascular Development and Repair Department, Centro Nacional de Investigaciones Cardiovasculares (CNIC), Melchor Fernindez Almagro 3, Madrid, Spain; 6 Parco Scientifico e Tecnologico, Stradale Vincenzo Lancia, 57, Catania, Italy; 7 Correspondence can be also addressed to giugno@dmi.unict.it

**Keywords:** Database, Genomic Information, Data Analysis, miRNA-Gene Expression, Pathway

## Abstract

**Background:**

*Vitis vinifera *(Grapevine) is the most important fruit species in the modern world. Wine and table grapes sales contribute significantly to the economy of major wine producing countries. The most relevant goals in wine production concern quality and safety. In order to significantly improve the achievement of these objectives and to gain biological knowledge about cultivars, a genomic approach is the most reliable strategy. The recent grapevine genome sequencing offers the opportunity to study the potential roles of genes and microRNAs in fruit maturation and other physiological and pathological processes. Although several systems allowing the analysis of plant genomes have been reported, none of them has been designed specifically for the functional analysis of grapevine genomes of cultivars under environmental stress in connection with microRNA data.

**Description:**

Here we introduce a novel knowledge base, called BIOWINE, designed for the functional analysis of *Vitis vinifera* genomes of cultivars present in Sicily. The system allows the analysis of RNA-seq experiments of two different cultivars, namely Nero d'Avola and Nerello Mascalese. Samples were taken under different climatic conditions of phenological phases, diseases, and geographic locations. The BIOWINE web interface is equipped with data analysis modules for grapevine genomes. In particular users may analyze the current genome assembly together with the RNA-seq data through a customized version of GBrowse. The web interface allows users to perform gene set enrichment by exploiting third-party databases.

**Conclusions:**

BIOWINE is a knowledge base implementing a set of bioinformatics tools for the analysis of grapevine genomes. The system aims to increase our understanding of the grapevine varieties and species of Sicilian products focusing on adaptability to different climatic conditions, phenological phases, diseases, and geographic locations.

## Background

*Vitis vinifera *[1] is one of the most important plant species in the modern world. Its economic and cultural impact is undeniable. With about 8 million hectares of vineyards across the world [2] and more than 5000 estimated cultivars [3], *Vitis vinifera *contributes significantly to the economy of wine-producing countries.

Improving the quality of wine and increasing the resistance of grapevine to pathogens and environmental stress are crucial tasks for the development of wine industry [4]. A deeper understanding of the underlying mechanisms can aid plantation development and effective pest and pathogen treatment design. This is particularly helpful when environmental conditions are not optimal (e.g. scarcity of water, climate instability). Finally, it drives the development of superior germplasm and the establishment of more robust and high-quality cultivars.

Recently, several projects have been devoted to acquire more knowledge about grapevine at a genomic and biomolecular level. A recent review of the cutting-edge of grape and wine biotechnologies can be found in [5]. In 2007, the French-Italian Public Consortium for Grapevine Genome Characterization presented the first sequenced genome of *Vitis vinifera *[6]. This was obtained from a highly homozygous genotype, by a total of 6.2 million end-reads, representing an 8.4-fold coverage of the grape genome. At a later stage, a higher quality sequenced genome (12-fold coverage), from highly heterozygous cultivar, was presented [7] and deposited in PlantGDB (http://www.plantgdb.org/VvGDB/). The availability of the whole grapevine genome, and its comparison with other available plant genomes (*Arabidopsis, Oryza sativa, Populus, Medicago *and *Solanum lycopersicum*), has led to the discovery of ancestral traits and genetic organization of flowering plants. Furthermore, the availability of the grape genome has driven a multitude of studies on the genetics of grapes, including genome annotation and the development of genomic resources such as bacterial artificial chromosomes (BAC) and cDNA libraries [8-10]. *Vitis vinifera *genes are associated with many existing pathways [11], concerning Metabolism, Genetic and Environmental Information Processing, Cellular Processes, Transport, and Transcription Factors. On the other hand, for several genes the functional annotation is still missing. Among the 29,971 known *Vitis vinifera* genes, more than 30% are without any annotation. Sequencing of grape genomes enables new studies on grapevine variety, aimed at the identification of the genetic origin of phenotypic traits, resistance and wine quality. Among the others, two genes have been associated with grape berry coloring [12], giving us evidence that white grapes were probably caused by the simultaneous mutation of these two adjacent regulatory genes. A comprehensive study of this hypothesis has been performed by Velasco et al [13]. They assembled de novo the genome of the heterozygous *Vitis vinifera *cultivar Pinot Noir and identified several genes involved in pathogen resistance and polyphenol metabolism (influencing vine quality). They also discovered about 2 million SNPs and mapped most of them to chromosomes. Later on, Myles et al [14] performed a large-scale polymorphism detection on 10 cultivars (including Pinot Noir, White Riesling and Malvasia, among others) and 7 wild *Vitis *species. They identified hundreds of thousands of SNPs and produced a 9K genotyping array, able to discriminate different cultivars. Laucou et al [15] performed a large study of genetic diversity involving 4,370 accessions of *Vitis* originating from different geographic areas. They used 20 simple sequence repeat markers leading to the identification of 524 alleles and 2,836 different profiles.

Considerable attention has been dedicated to the analysis of the grapevine transcriptome. Studies on gene expressions and trascriptional profiling have shed light on several biological processes of *Vitis vinifera *involving ripening and maturation [16-19], dormancy transitioning [20], and resistance to pathogens and environmental conditions [21-23]. High throughput sequencing approaches has led to characterizing various transcripts of *Vitis*, in particular microRNAs, small RNA molecules that have been shown to play a central role in gene regulation within several important biological processes. Mica et al. [24] used oligonucleotide arrays to measure the expression of previously identified putative microRNAs in different tissues and during fruit maturation. They found several patterns of differentially expressed microRNAs and identified candidate splicing events. Wang et al [25] built a library of small RNAs of *Vitis amurensis*, a resistant wild type of *Vitis *genus, and identified several conserved and non-conserved microRNAs.

Currently, *Vitis vinifera*'s genomic data are stored by multiple sources, each of them maintaining a specific kind of data (genomic [26-28], miRNA [24], SNPs [13] and pathways [11]).

VitisNet [11] stores manually annotated biological networks of *Vitis Vinifera *and allows the understanding of dynamic processes in systems biology experiments. All VitisNet files together with the manual annotation of the grape genome encompassing pathway names, individual genes, their genome identifier, and chromosome location can be accessed and downloaded from the VitisNet portal.

Recently, in [29] authors proposed VTCdb, a grapevine gene co-expression database equipped with basic functional enrichment and visualization capabilities. This computational tool allows querying RNA-Seq data for grapevine berry development [30].

Here we present BIOWINE, a novel platform to manage and analyze genomic data of *Vitis vinifera*. BIOWINE is a free web-based resource allowing users to browse and query *Vitis vinifera *RNA-seq data properly integrated with knowledge coming from different external sources including genes, transcripts, proteins, microRNAs, pathways and Gene Ontology (GO) associations.

BIOWINE is complementary to the *Vitis *data sources, such as VitisNet and VTCdb, and allows a more comprehensive genomic analysis of *Vitis Vinifera *cultivars. It integrates data from multiple sources and enables for comprehensive genomic analysis of grapevine cultivars at different phenological and pathological states. It allows the analysis of novel data obtained with state-of-the-art RNA-seq. BIOWINE computes the abundance of transcripts from RNA-seq data, obtaining highly reliable expression data for both mRNAs and short non-coding RNAs such as microRNAs. Data and results can be properly visualized and downloaded through an appropriate web-interface available at the following URL: http://alpha.dmi.unict.it/biowine/.

## Construction and content

### Data source

*Vitis Vinifera samples*. BIOWINE is a knowledge base storing RNA-seq data of *Vitis vinifera *native Sicilian grapevines in different climatic and phenological conditions. The samples have been selected both healthy and with diseases such as water stress, leaf curling, iron chlorosis and yellow mosaic.

**Figure 1:**
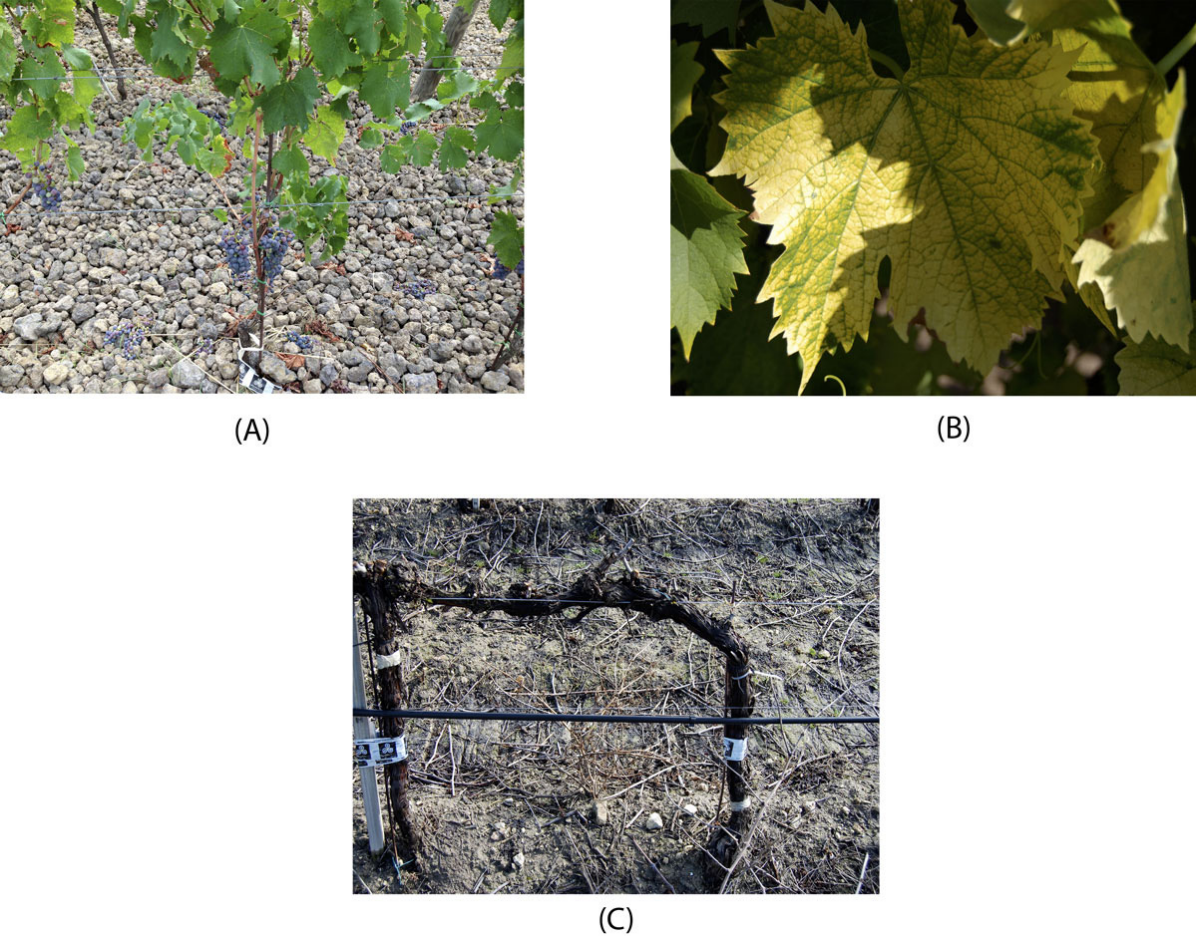
**(A) A plant presenting a water stress condition**. (B) A plant presenting yellow Mosaic disease. (C) Woody tissues used for sampling the water stressed grapevine.

The presence of water stress was inferred by carrying out a recognition the year before sampling. The plants to be sampled affected by water stress have been pointed by the technical director of the company on the basis of the ground with excessive skeleton (see. Figure 1 (A)) and water stress presented each year. Yellow mosaic is caused by chromogenic grapevine fan-leaf nepovirus (GFLV) strains. Affected vines show chrome-yellow discolorations that develop early in the spring and may affect all vegetative parts of the vines (leaves, herbaceous shoot axes, tendrils and inflorescences, see Figure 1 (B)). Chromatic alterations of the leaves vary from a few scattered yellow spots, sometimes appearing as rings and lines, to variously extended mottling of the veinal and/or interveinal areas, to total yellowing. In spring, affected plants in a vineyard can readily be spotted from a distance. Malformations of leaves and canes are usually not prominent, but clusters may be smaller than normal and may have shot berries. In hot climates the newly produced summer vegetation has a normal green colour, while the yellowing of the old growth turns whitish and tends to fade away. Our inspections identified the following plant material:

• Grapevine Nerello Mascalese located in the territory of Randazzo (CT), 672,8 m above sea level, planted in 2006. Inside the vineyard the following plants have been selected: 3 apparently healthy; 3 showing symptoms of leaf curling; 3 with clear water stress; 1 with Mosaic disease.

• Grapevine Nerello Mascalese located in the territory of Linguaglossa (CT), 614 m above sea level, planted in 2007. Inside the vineyard 3 apparently healthy plants and 1 with Mosaic disease have been selected.

• Grapevine Nero d'Avola located in the territory of Caltanissetta (CL), 379 m above sea level, planted in 2001. Inside the vineyard the following plants have been selected: 3 apparently healthy; 3 with obvious iron chlorosis.

• Grapevine Nero d'Avola located in the territory of Marzamemi (SR), 12 m above sea level, planted in 1989. Inside the vineyard 3 apparently healthy plants have been selected.

• Grapevine Nero d'Avola located in the territory of Noto (SR), 25 m above sea level, planted in 1996. Inside the vineyard 3 apparently healthy plants have been selected.

Within the identified plants it was decided to carry out the following sampling during different phenological stages:

• Vegetative sleeping: Sampling of woody shoots.

• Germination: Sampling of leaves.

• Bloom: Sampling of leaves.

• End of flowering: Sampling of berries.

• Bunch closure: Sampling of berries.

• Veraison: Sampling at ripening.

• Fruit: Sampling of berries.

For sampling at ripening, each biological replicate comprised a pool of berries from 3 to 5 grapes collected on the same plant. The collection of samples for the water-stressed vine during vegetative sleeping were taken from the woody tissues from vines pruned previously. These were pooled and subsequently subjected to RNA extraction (see. Figure 1 (C)).

See Table 1 for the complete list of all the sequenced samples.

**Table 1:** The list of sequenced samples.

Sample ID	Growing location	Grapevine	Phenotype	Phenological Phase	Age
CL-1	Caltanissetta	Nero D'Avola	Normal	Vegetative sleeping	12 years

CL-2	Caltanissetta	Nero D'Avola	Iron chlorosis	Vegetative sleeping	12 years

MA-1	Marzamemi	Nero D'Avola	Normal	Vegetative sleeping	24 years

NO-1	Noto	Nero D'Avola	Normal	Vegetative sleeping	17 years

LI-1	Linguaglossa	Nerello Mascalese	Normal	Vegetative sleeping	5 years

RA-1	Randazzo	Nerello Mascalese	Water stress	Vegetative sleeping	7 years

RA-2	Randazzo	Nerello Mascalese	Leaf curling	Vegetative sleeping	7 years

RA-3	Randazzo	Nerello Mascalese	Normal	Vegetative sleeping	7 years

MA-2	Marzamemi	Nero D'Avola	Normal	Germination	24 years

NO-2	Noto	Nero D'Avola	Normal	Germination	17 years

CL-3	Caltanissetta	Nero D'Avola	Normal	Germination	12 years

CL-4	Caltanissetta	Nero D'Avola	Iron chlorosis	Germination	12 years

RA-4	Randazzo	Nerello Mascalese	Water stress	Germination	7 years

RA-5	Randazzo	Nerello Mascalese	Leaf curling	Germination	7 years

RA-6	Randazzo	Nerello Mascalese	Normal	Germination	7 years

LI-2	Linguaglossa	Nerello Mascalese	Normal	Germination	5 years

MA-3	Marzamemi	Nero D'Avola	Normal	End of flowering	24 years

NO-3	Noto	Nero D'Avola	Normal	End of flowering	17 years

CL-5	Caltanissetta	Nero D'Avola	Normal	End of flowering	12 years

CL-6	Caltanissetta	Nero D'Avola	Iron chlorosis	End of flowering	12 years

LI-3	Linguaglossa	Nerello Mascalese	Normal	End of flowering	5 years

LI-MS1	Linguaglossa	Nerello Mascalese	Mosaic	End of flowering	5 years

RA-7	Randazzo	Nerello Mascalese	Water stress	End of flowering	7 years

RA-8	Randazzo	Nerello Mascalese	Leaf curling	End of flowering	7 years

RA-9	Randazzo	Nerello Mascalese	Normal	End of flowering	7 years

MA-4	Marzamemi	Nero D'Avola	Normal	Fruit	24 years

NO-4	Noto	Nero D'Avola	Normal	Fruit	17 years

CL-7	Caltanissetta	Nero D'Avola	Normal	Fruit	12 years

CL-8	Caltanissetta	Nero D'Avola	Iron chlorosis	Fruit	12 years

LI-4	Linguaglossa	Nerello Mascalese	Normal	Fruit	5 years

LI-MS2	Linguaglossa	Nerello Mascalese	Mosaic	Fruit	5 years

RA-10	Randazzo	Nerello Mascalese	Water stress	Fruit	7 years

RA-11	Randazzo	Nerello Mascalese	Leaf curling	Fruit	7 years

RA-12	Randazzo	Nerello Mascalese	Normal	Fruit	7 years

RA-MS2	Randazzo	Nerello Mascalese	Mosaic	Fruit	7 years

MA-5	Marzamemi	Nero D'Avola	Normal	Bunch closure	24 years

NO-5	Noto	Nero D'Avola	Normal	Bunch closure	17 years

LI-5	Linguaglossa	Nerello Mascalese	Water stress	Bunch closure	5 years

LI-MS3	Linguaglossa	Nerello Mascalese	Mosaic	Bunch closure	5 years

RA-13	Randazzo	Nerello Mascalese	Water stress	Bunch closure	7 years

RA-14	Randazzo	Nerello Mascalese	Leaf curling	Bunch closure	7 years

RA-15	Randazzo	Nerello Mascalese	Normal	Bunch closure	7 years

RA-MS3	Randazzo	Nerello Mascalese	Mosaic	Bunch closure	7 years

CL-9	Caltanissetta	Nero D'Avola	Normal	Bunch closure	12 years

CL-10	Caltanissetta	Nero D'Avola	Iron chlorosis	Bunch closure	12 years

CL-11	Caltanissetta	Nero D'Avola	Normal	Veraison	12 years

LI-6	Linguaglossa	Nerello Mascalese	Normal	Veraison	5 years

LI-MS4	Linguaglossa	Nerello Mascalese	Mosaic	Veraison	5 years

RA-16	Randazzo	Nerello Mascalese	Water stress	Veraison	7 years

RA-17	Randazzo	Nerello Mascalese	Leaf curling	Veraison	7 years

RA-18	Randazzo	Nerello Mascalese	Normal	Veraison	7 years

*RNA isolation*. Total RNAs for mRNA-seq were extracted using Spectrum Plant Total RNA Kit (Sigma-Aldrich) following the manufacturer's instructions. MiRNA/small RNAs were purified with MirPremier microRNA Isolation Kit (Sigma-Aldrich) following the manufacturer's instructions. The starting materials were tissues conserved in RNALater Stabilization Solution (Ambion).

*RNA-sequencing*. RNA samples were processed using TruSeq mRNA-seq sample prep kit from Illumina (Illumina, Inc., CA, USA). Briefly, the poly-A containing mRNA molecules were purified using poly-T oligo-attached magnetic beads, fragmented into small pieces using divalent cations under elevated temperature, cDNA was synthesized by reverse transcription and standard blunt-ending plus add 'A' was performed. Then, Illumina TruSeq adapters with indexes were ligated to the ends of the cDNA fragments. After ligation reaction and separation of not ligated adapters, samples were amplified by PCR to selectively enrich those cDNA fragments in the library having adapter molecules at both ends. Each 6-plex pool was sequenced on HiSeq2000 at Institute of Applied Genomics (Udine, Italy), producing about 20 million of single-reads per sample, 50 bp long.

*miRNA-sequencing*. RNA samples were processed by using TruSeq smallRNA-seq Sample Prep kit from Illumina (Illumina, Inc., CA, USA), following the subsequent steps. First the sequential ligation of the RNA 3' and RNA 5' adapters to the samples was performed, then the reverse transcription was followed by PCR to selectively enrich those cDNA fragments in the library having adapter molecules at both ends. Individual libraries with unique indices were pooled in 12plex and the bands corresponding to approximately the adapter-ligated constructs derived from the 22 nt and 30 nt small RNA fragments were recovered from a 6% PAGE gel. Each 12-plex pool was sequenced on HiSeq2000 at Institute of Applied Genomics (Udine, Italy), producing about 10 million of single-reads per sample, 50 bp long.

*RNA-seq analysis*. In order to analyze RNA-seq data, a standard pipeline has been adopted. This is based essentially on the well-known Tophat2/Cufflinks/Cuffdiff pipeline by Trapnell et al [31]. As the first step, raw sequences have been trimmed. Then, reads of each sample have been aligned against the reference genome and finally the alignments have been processed by Cufflinks which is able to compute transcript abundances in Fragments Per Kilobase of exon per Million fragments mapped (FPKM). This allowed for obtainment of gene expression levels for each sample. In order to perform a differential expression analysis between each pair of samples in each phonological phase, the log2 fold change of FPKMs was computed by using Cuffdiff.

Notice that Cufflinks has been used only for computing abundance of known transcripts. Future research work includes the possibility of analyzing putative novel transcripts (by running Cufflinks and Cuffmerge packages after Tophat2 and before Cuffdiff).

We also indexed all the putative SNPs and INDELs found in the sequenced genomes. The pipeline used for Variant calling has been based on Samtools [32].

*miRNA analysis*. To remove adapter sequences, we trimmed Raw sequence by a proprietary script. Therefore, we considered only putative small RNA sequences. Next, we produced a list of unique sequences (tags) together with their occurrences. Each unique sequence was then mapped against the corresponding miRBase entry to build a table of counts of each known miRNA in each sample. Finally, the counts table was normalized according to the DESeq [33] normalization algorithm implemented in the DESeq Bioconductor package. As results, we obtained a table of miRNA expression levels.

Moreover, in order to discover new putative miRNAs, small RNA sequences have been processed by using the miRDeep-P [34] package which is able to identify miRNA in plant species. Each sample was processed and a set of predicted miRNAs that meets the criteria of plant miRNAs was given in output. These new putative miRNAs will be investigated for a validation in a future work.

*Integrated knowledge bases*. The genomic information have been integrated with third party databases. Genes have been associated with their GO terms through the Ensembl Biomarts [35], miRNAs were annotated with information about their precursor and mature sequences coming from miRBase [36]. Pathways have been obtained from KEGG [37]. We used UNIPROT [38] to index protein sequences. Finally, from psRNATarget [39] we stored information about microRNAs targets.

### Database schema and implementation

The BIOWINE knowledge base has been built and maintained up-to-date on top of a MySQL database management system, accessible through the lightweight interface PHP Data Objects running on an Apache server. The front-end web has been developed by using Bootstrap and HTML. The database contains sixteen tables to store genes, samples, SNPs, validated and predicted miRNAs, mRNAs, coded proteins, pathways and GO terms. The system has been implemented by adopting a Model-View-Controller approach. The processing of the results as well as the extraction of data from the database are made by using Python and R. BIOWINE relies on Python scripts for enrichment and on R scripts for generating heatmaps (pheatmap library) and volcano plots (ggplot2 library). Each submitted job is associated with a unique alphanumeric identifier allowing subsequent consultations. Finally, two levels of controls on the input provided by the user have been implemented, a client-level and a server-level, in order to guide the writing and to avoid misspelling input.

## Utility and Discussion

### The BIOWINE Interface

BIOWINE is free web based resource allowing users to browse and query *Vitis vinifera *RNA-seq data. BIOWINE implements a custom version of genome browser GBrowse [40] for *Vitis vinifera *genome 12x available from [26].

All the functional annotation tools can be accessed from the home page of BIOWINE.

**Figure 2:**
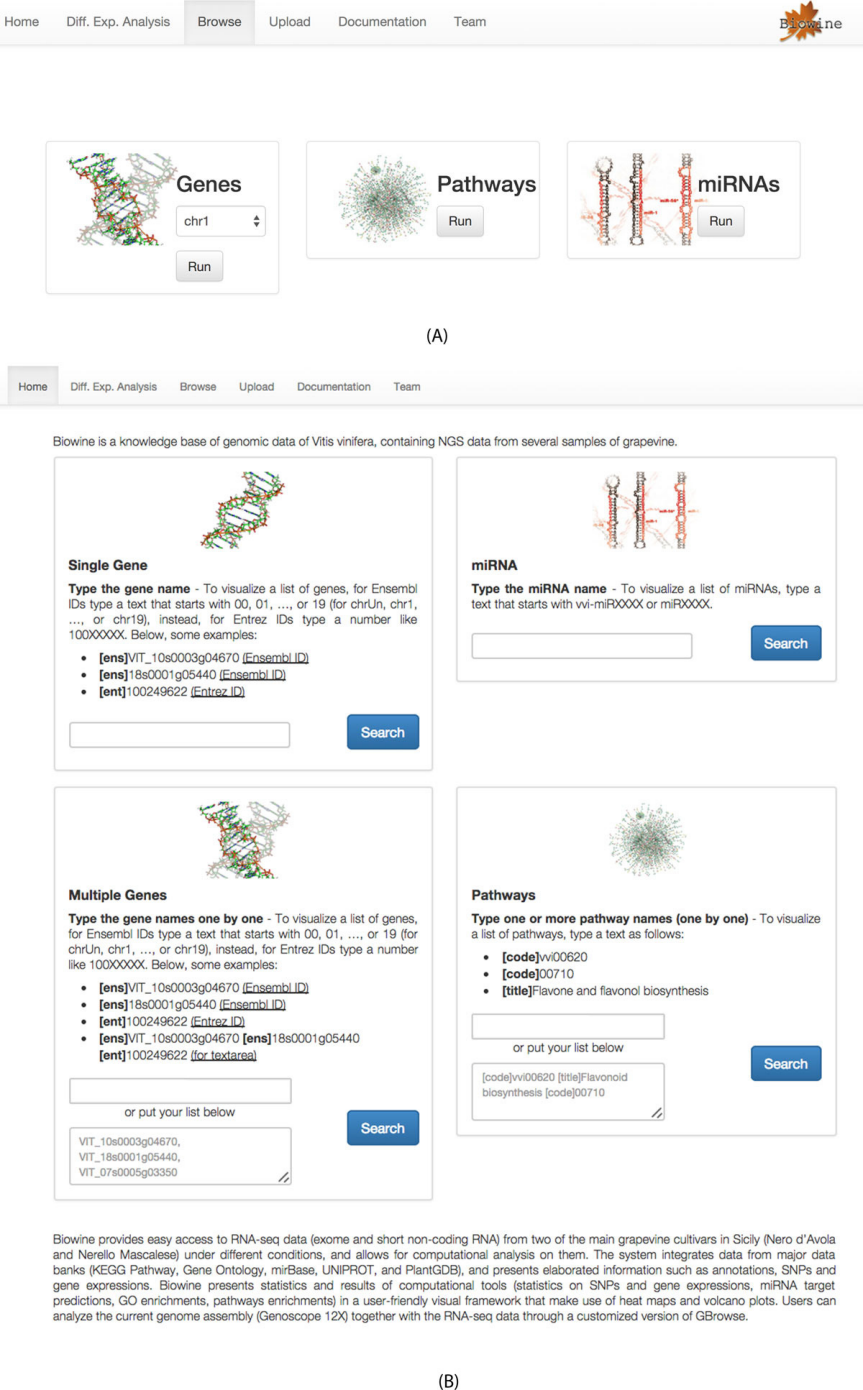
**BIOWINE main interface**. (A) User lists all genes by chromosome, pathways, and miRNAs. (B) User searches information by (i) a single gene, miRNA, and pathway, or (ii) shared among a list of genes or pathways.

*Browsing*. User can access all the data stored within BIOWINE according to a 'subject' which can be genes, pathways or microRNAs (see Figures 2 (A)).

*Search by single gene or miRNA*. A simple search is used to get genomic and quantitative information about a single gene within the reference genome as observed by our RNA-seq experiments (see Figure 2 (B)).

**Figure 3:**
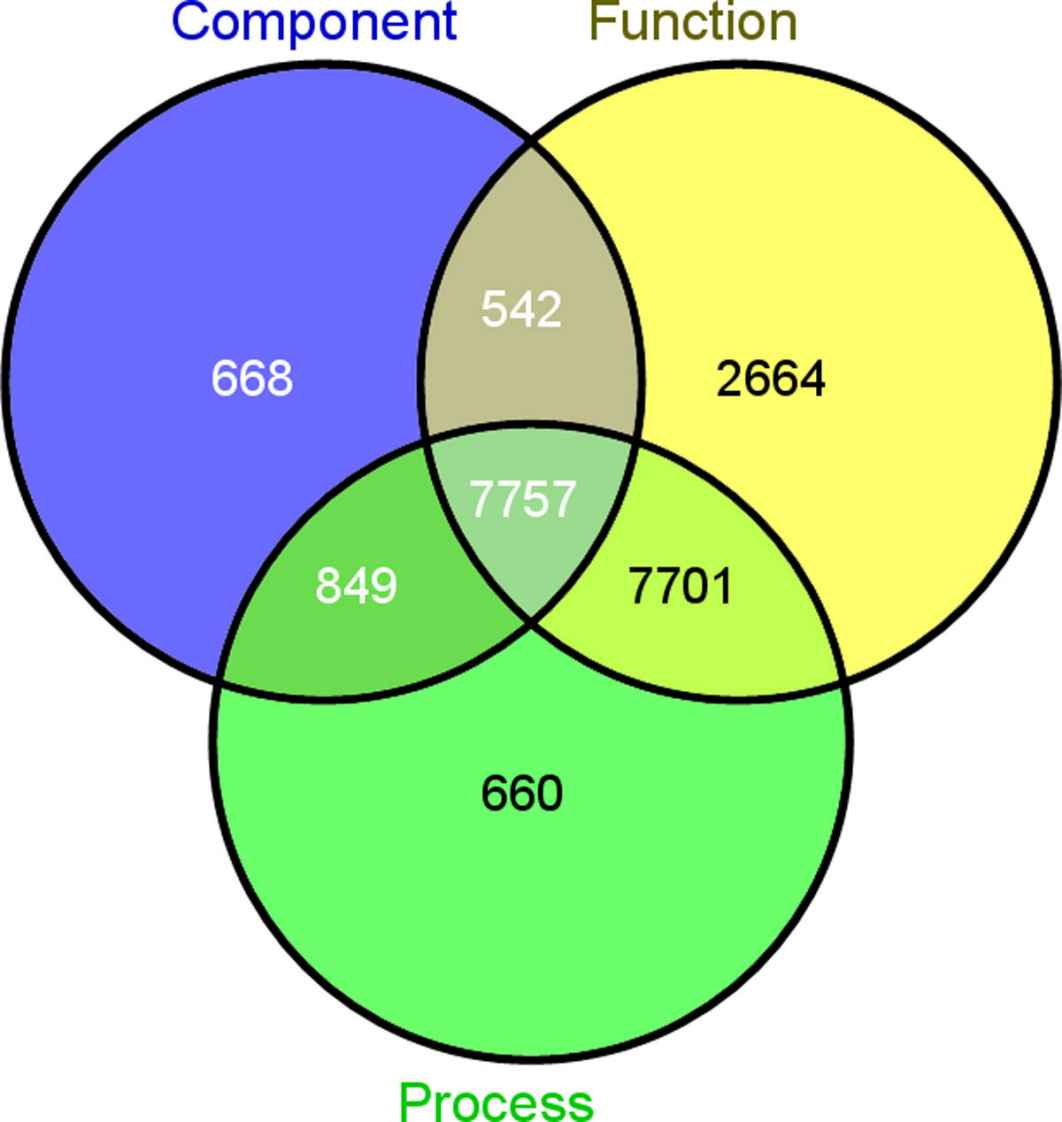
**Distribution of genes GO annotations**. Among 29971 genes present in BIOWINE, we have 16967 genes annotated with a biological process; 18664 genes annotated with a molecular function; 9816 genes annotated with a cellular component. More than 30% of genes (9130) are without any annotation.

Once the user types the query, the system yields a page with all the information associated with that gene. The page contains several sections. The first one shows the chromosome location, the strand, the starting and ending position together with the NCBI and EBI gene IDs if known. A link to GBrowse allows to view the gene within the BIOWINE genomic browser. A second section gives the list of associated GO Terms (in Figure 3 we report the distribution of GO annotation in our knowledge base). The complete mRNA sequence together with the 3' and 5' UTR regions are given. A subsection within the mRNA panel gives an exonview capability of the gene expression for each sample. Through such a panel the user analyzes the expression level of each single exon in each sample.

The next section lists all the known microRNAs targeting such a gene. Also in this case a link to GBrowse shows each miRNA within the BIOWINE genomic browser. Then, we report all the known proteins encoded by such a gene. Next we list all the pathways in which the gene enters. Then, BIOWINE gives a section with all the SNPs and INDELs observed in our sequenced samples. For each sample BIOWINE gives the type of event (SNP or INDEL), the nucleotide in the reference genome and the observed one. Information about SNPs and INDELs are given together with a "Quality" value which is basically a measure of how confident Samtools [32] are about that variant. The Genotype Quality (GQ) value encodes the read quality score. The user can limit the visualized SNPs by choosing the proper range of GQ values. The GQ range can be specified through the dedicated combo boxes at the beginning of the section.

Next, we report a heatmap with the fold change of the gene expression for each pair of sequences samples. We show only the pairs of samples having a fold change supported by statistical significance. We also give a volcano plot of the results.

Finally, through the last section the user can download all data in .csv and .txt formats.

Similarly, through the searching by a single miRNA, BIOWINE shows all targeting information (genes and MRE (MiRNA Recognition Elements)) together with its expression over the samples.

*Search by multiple genes*. The search by multiple genes works by pasting a list of genes (see Figure 2 (B)). The search can be done either by gene names (e.g. VvMYBA1) or by external identifiers from NCBI or EBI.

BIOWINE performs a functional analysis on them. The system yields a page in which the user can find the genomic information of each gene.

The results page is organized in the following sections.

**Figure 4:**
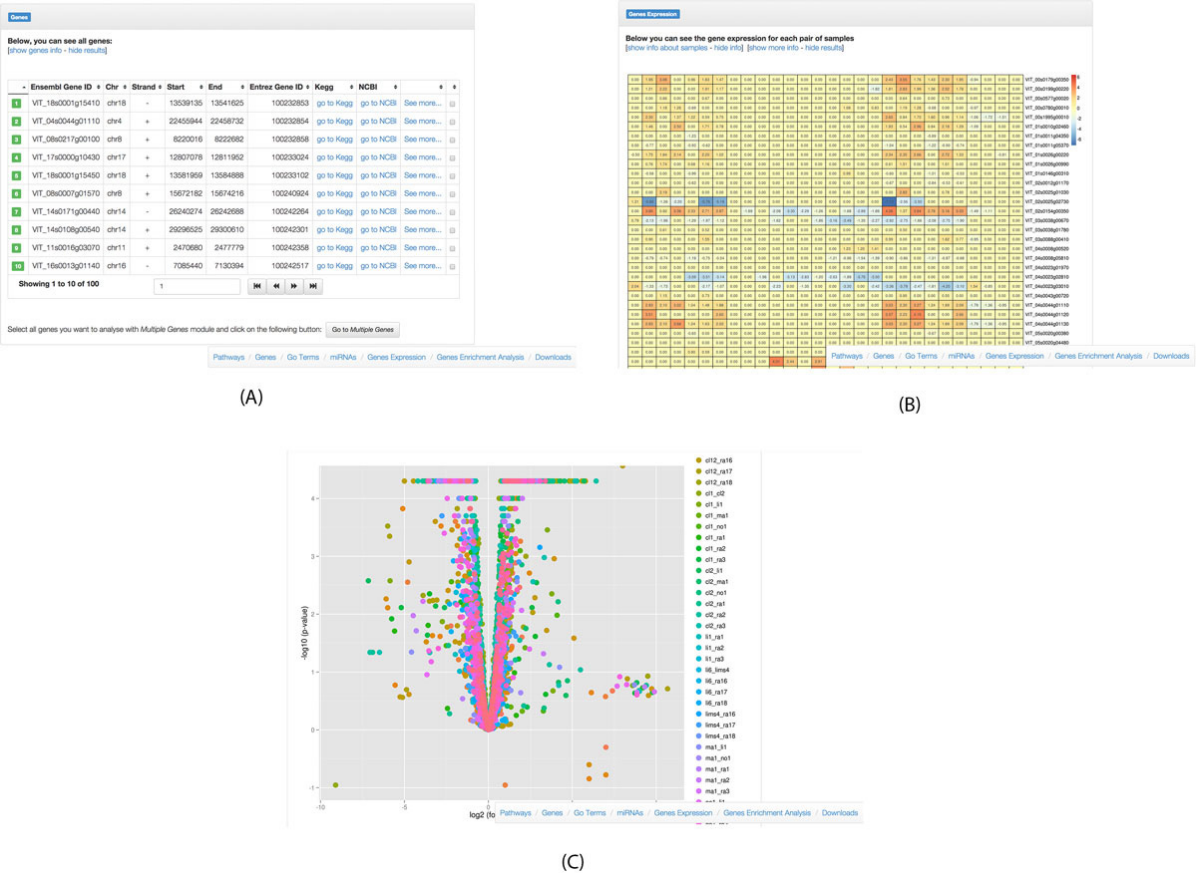
**(A) The basic genomic information of the genes given as input; (B) Sample pair comparisons of gene expressions; (C) A volcano plot is also shown**. Notice that the figure partially reports the result page. By scrolling the result page, users can jump to any of the following sections: Pathways, Genes, Go Terms, miRNAs, Gene expression, Gene enrichment Analysis, Downloads. By using the bottom right menu, users can download results in .csv format.

The "gene" panel summarizes the basic genomic information of the genes given as input (see Figure 4 (A)). For each gene BIOWINE gives a link to the gene details pointing to a BIOWINE page with the single gene information. Next, a list of enriched GO terms, if any, is given. The enrichment level of a gene set is evaluated through Fisher's exact test with a default cutoff of 0.05. We also apply the Bonferroni test for multiple testing correction.

Then, a section lists the microRNAs targeting those a genes. The section showing the list of enriched pathways follows. Finally we provide a heatmap with the fold change of each pair of samples (see Figure 4 (B)). For each of them we give a table with the genes having a statistically significant fold change. We report also the volcano plot of the fold change values (see Figure 4 (C)).

Finally, the download section allows users to store locally all the functional analysis results in a text format.

*Search by pathways*. BIOWINE stores *Vitis Vinifera *pathway details obtained from KEGG. Users can query the system by giving as input the code or the title of one or more pathways (see Figure 2 (A)),

BIOWINE performs a functional enrichment on these. The system yields the list of genes shared by those pathways. Then, a section lists the enriched GO Terms. The next panel gives the microRNAs targeting those genes if any. Then a list with the pathways in which those genes enter is given. Next, the heatmap with the fold change of each pair of samples is given. The download section allow to store locally all the data.

*Comparative differential expression analysis*. Here, user is able to perform comparative analysis on the genes/miRNAs expression of all the RNA-seq data stored in BIOWINE. It is possible to compare one or more subjects among phenotype, phenological phase, grapevine, growing location and sample ID. The result page shows a heatmap with the genes/miRNAs having a statistical significant differential expression in the compared classes together with the list of enriched GO terms and pathways.

*Network visualization of results*. Cytoscape web network visualization (http://www.cytoscape.org/) has been used to show the protein-protein interactions related to the searched genes or the genes contained in the searched pathways and their neighbors together with all miRNAs targeting such genes. When searching by miRNA, BIOWINE shows the interactions among the miRNA targeting genes and their adjacent PPI.

*Case Study*. In what follows we present a BIOWINE investigation inspired by known results in the literature. This is an example of considerations that may be conducted through BIOWINE by analyzing phenotype and genomic data.

In [41] Wang et al. studied the VvWRKY proteins which are an important class of transcriptional regulators in higher plants. Through a bioinformatics pipeline authors characterized the large class of WRKY genes in *Vitis vinifera*. Then, members of such a VvWRKY family genes were grouped into three main classes according to their exon-intron structures and motif compositions. Authors identified two subclasses containing genes exhibiting different patterns of expression in response to different stresses. We selected such a list of genes as reported in Table 2 and ran the functional analysis through BIOWINE. The analysis highlights that VIT 09s0018g00240 is over expressed on CL-2 sample which is subject to Iron chlorosis, and it presents SNPs only on such a sample. VIT 16s0050g02510 is clearly downregulated on all Nerello Mascalese samples. In particular on RA-1, which is subject to water stress, VIT 16s0050g02510 presents a deletion in the transcript. In Table 3 we report the enriched GO terms of the vVwRKY selected proteins. We can notice that genes VIT 04s0008g05750 and VIT 09s0018g00240 are enriched by the term "response to salicylic acid" which alleviates decreases in photosynthesis under heat stress and helps the recovery in grapevine leaves [42].

**Table 2:** Selected genes for the case study.

Genes	Ensable gene ID	chr
1	VIT 04s0008g05750	4

2	VIT 09s0018g00240	9

3	VIT 02s0025g01280	2

4	VIT 08s0058g01390	8

5	VIT 13s0067g03140	13

6	VIT 15s0046g01140	15

7	VIT 16s0050g02510	16

**Table 3:** Case study: Enriched Process and Functions

GO Term	Description	p-value	Genes
Processes			

GO:0006355	regulation of transcription, DNA-templated	8.56e-09	1 2 3 4 5 6 7

GO:0050691	regulation of defense response to virus by host	1.46e-07	1 2

GO:0031347	regulation of defense response	9.65e-05	1 2

GO:0002237	response to molecule of bacterial origin	0.0001	1 2

GO:0009751	response to salicylic acid	0.0004	1 2

Functions			

GO:0043565	sequence-specific DNA binding	3.82e-13	1 2 3 4 5 6 7

GO:0001071	nucleic acid binding transcription factor activity	1.49e-10	1 2 3 4 5 6 7

GO:0003700	sequence-specific DNA binding transcription factor activity	1.6e-10	1 2 3 4 5 6 7

GO:0003677	DNA binding	3.08e-08	1 2 3 4 5 6 7

Enriched process and functions.

## Conclusions

BIOWINE is a web resource for the study of *Vitis vinifera *genome. It stores exome and small RNAs RNA-seq sequencing data of two Sicilian grapevines: Nerello Mascalese e Nero D'avola. Its flexible web interface allows for the functional analysis of gene and microRNA expression, SNPs and exome expression variations of grapevine samples on different phenological phases and diseases status. BIOWINE integrates third party databases concerning GO Terms and pathways and implements a custom version of GBrowse with the *Vitis Vinifera *genome 12x. BIOWINE will be maintained up-to-date and it will integrate *V. Vinifera *genome data of other grapevines present in Italy.

## Competing interests

The authors declare that they have no competing interests.

## Authors' contributions

AP, RG and AF conceived the research. AP and RG developed and coordinated the research. RD and GP designed and implemented the system. MM and GG developed the functional enrichment module. VV, AL and FC provided and analyzed the samples. AP, RG, RD, GP, VV, AL, FC and AF contributed to analysis aspects and wrote the paper.

## Declarations

Publication of this article has been funded by PON 2007-2013 grant, SIGMA - PON01 00683 - CUP B61H11000380005.

This article has been published as part of *BMC Systems Biology *Volume 9 Supplement 3, 2015: Proceedings of the Italian Society of Bioinformatics (BITS): Annual Meeting 2014: Systems Biology. The full contents of the supplement are available online at http://www.biomedcentral.com/bmcsystbiol/supplements/9/S3.
